# Dr. Morey C. Collier

**Published:** 1917-10

**Authors:** 


**Dr. Morey C. Collier,** Albany 1906, died suddenly at his home in Painted Post, Sept. 15, of cerebral haemorrhage.

